# Trust but Verify: Discordance in Opioid Reporting Between the Electronic Medical Record and a Statewide Database

**DOI:** 10.7759/cureus.31027

**Published:** 2022-11-02

**Authors:** Amit Pujari, Mili Patel, Aria Darbandi, John Garlich, Milton Little, Carol Lin

**Affiliations:** 1 Orthopedic Surgery, Cedars-Sinai Medical Center, Los Angeles, USA; 2 Medicine, Icahn School of Medicine at Mount Sinai, New York, USA; 3 Orthopedics, Cedars-Sinai Medical Center, Los Angeles, USA

**Keywords:** orthopaedics, trauma, pain management, opioids, pain

## Abstract

Introduction: The electronic medical record (EMR) is often used as the primary source for patient medication lists and history. We sought to determine the accuracy of the EMR in documenting opioid prescriptions in patients undergoing fracture repair compared to a statewide database.

Methods: This retrospective study was conducted at an urban level 1 trauma center. Patients > 18 years old were included if they were admitted directly through the emergency room with isolated single orthopedic injuries. Opioid use and prescription data prior to admission and three months following surgery were collected through the EMR and a California statewide database of controlled substance prescriptions. A 2 x 2 McNemar’s test was used to identify discordance between the EMR and Controlled Substance Utilization Review and Evaluation System (CURES).

Results: A total of 369 patients were included. The EMR reported that 143 patients had an opioid prescription within 30 days prior to admission compared to 75 patients reported by CURES (discordance rate [DR]: 34.7%) (p < 0.001). Between postoperative days (POD) 0-30, the EMR reported that 367 patients had an opioid prescription compared to 285 reported by CURES (DR: 22.8%) (p < 0.001). Between POD 30-60, the EMR reported that 142 patients had an opioid prescription compared to 84 reported by CURES (DR: 34.7%) (p < 0.001). Between POD 60-90, the EMR reported that 83 patients had an active opioid prescription compared to 60 patients reported by CURES (DR: 41.0%) (p = 0.10).

Conclusion: There is a significant discordance between the databases in documenting opioid use. Physicians should check multiple sources to best assess active opioid prescriptions.

## Introduction

Americans consume approximately 80% of the global opioid supply and nearly 99% of the global hydrocodone supply [1]. Prior research has shown orthopedic surgeons are one of the highest prescribers of opioids among all specialties in the United States [2]. Opioids often play a significant role in pain management following orthopedic surgery and injury, but a balance must be obtained between providing appropriate pain management for patients while reducing the risk of opioid addiction and complications [3]. Since 1999, opioid overdoses have nearly tripled with opioid prescribing habits increasing in parallel [3]. Opioid prescriptions have also been found to be associated with an increased risk for postoperative complications including thromboembolic, infectious, and gastrointestinal complications [4].

Opioid prescription habits are often recorded in both a medical institution’s electronic medical record (EMR) and, if available, a statewide prescription drug monitoring program (PDMPs). The EMR records opioids that are prescribed and what the patient reports in their history as compared to PDMPs that record prescription utilization (when opioid prescriptions are filled). In order to decrease aberrant opioid use and opioid prescribing practices, PMDPs have been implemented by 49 states, including California [5]. The Centers for Disease Control and Prevention (CDC) denotes that PDMPs serve to be one of the most effective state-level interventions to improve opioid prescribing habits and monitoring [5].

The use of the PDMP offers a comprehensive record of opioid prescriptions being filled for a patient and offers the potential to prevent “doctor shopping” from occurring. However, the EMR is often used as the primary source for patient medication lists and history during a patient interaction which, in turn, influences future prescribing decisions. While most states recommend, or even mandate, that providers check PDMPs before prescribing opioids, the actual practice is not universal, and many prescribers rely primarily on the EMR to assess a patient’s opioid usage [6]. Furthermore, while the PDMP may capture opioid prescriptions written for that patient, it may not capture recreational use or if a patient is taking medications prescribed for someone else, which may be more likely to be captured in the EMR via patient history or self-report. As such, understanding how accurately the EMR reflects a patient’s opioid use may improve patient safety. To our knowledge, there have been no studies comparing the accuracy of a hospital EMR to a statewide database of opioid prescriptions. We sought to determine the accuracy of the EMR in documenting opioid prescriptions following orthopedic fracture repair compared to our statewide controlled substance database.

This article was previously presented as a meeting abstract at the 2020 Western Orthopedic Association Annual Meeting on August 2, 2020. 

## Materials and methods

Ethics approval

This study was approved by the local institutional review board. Patients between July 2018 and May 2019 who presented to an urban level-one trauma center with a fracture of the pelvis or extremities excluding the hand requiring operative fixation were included in this retrospective study.

Population

Patients were included if they were above 18 years of age at the time of surgery, received orthopedic operative fixation for a fracture, and if they were direct admission via the emergency department into the hospital for immediate operative fixation. Patients were excluded if they were initially seen by an outside provider for their fracture or had a re-operation within 90 days; these exclusion criteria were set to reduce the likelihood of patients having a prescription outside of the institutional EMR and to more accurately track opioid use associated with a single injury. Patients were also excluded if they were hospitalized for more than 30 days, were lost to follow-up before 90 days, or if they did not have 90 days of Controlled Substance Utilization Review and Evaluation System (CURES) data following their surgery to capture all follow-up time points.

Outcomes

The primary outcome of this study was the discrepancy observed between cohorts in the different time periods of preoperative and postoperative opioid use abstracted from both the EMR and CURES. CURES is a California statewide database to document the administration of controlled substances throughout the state which maintains records for one year. Secondary outcomes included observed opioid administration rates cumulatively between both databases. A trained research fellow collected and managed the opioid administration data.

Opioid use in the EMR was defined as what was reported in the patient’s outpatient medication list at the time of hospital admission for fracture and subsequent orthopedic outpatient follow-up, which are typically updated by pharmacists, nurses, the admitting physician for hospital admission, and the orthopedic surgeon during follow-up.

Patient opioid prescriptions were examined and recorded from both databases in a binary (yes/no) method. Patient records were examined for opioid use within 30 days prior to surgery (one month prior), 0-30 days following surgery, 31-60 days following surgery, and 61-90 days following surgery (three months postoperatively).

Discrepancies between the EMR and CURES for each aforementioned time period were calculated using a 2 x 2 McNemer test. A discrepancy was defined as only one of the two databases having a record of opioid administration during a given period. Statistically significant discrepancy rates were any discrepancy with a p-value < 0.05. Secondary outcomes included the opioid administration rate observed through each period by each database. All statical analyses were done by a trained statistician using RStudio [7].

## Results

A total of 942 patients received operative fixation for a fracture by the orthopedic trauma team between July 2018 and May 2019. A total of 369 patients met both inclusion and exclusion criteria and were included in this study.

According to the EMR (Table 1), 143 patients (38.8%) had an active opioid prescription prior to their surgery; 367 patients (99.5%) had an active opioid prescription between postoperative days 0-30, 142 patients (38.5%) had an active opioid prescription between postoperative day 31-60. Lastly, 83 patients (22.5%) had an active opioid prescription between postoperative days 61-90.

**Table 1:** CURES versus EMR: cumulative opioid usage between databases CURES: Controlled Substance Utilization Review and Evaluation System; EMR: Electronic medical record.

Postoperative Days	Opioid Usage CURES (Patients)	Opioid Usage EMR (Patients)
Prior to admission	75 (20.3%)	143 (38.8%)
0-30	285 (77.2%)	367 (99.5%)
31-60	84 (22.8%)	142 (38.5%)
61-90	60 (16.3%)	83 (22.5%)

**Figure 1:**
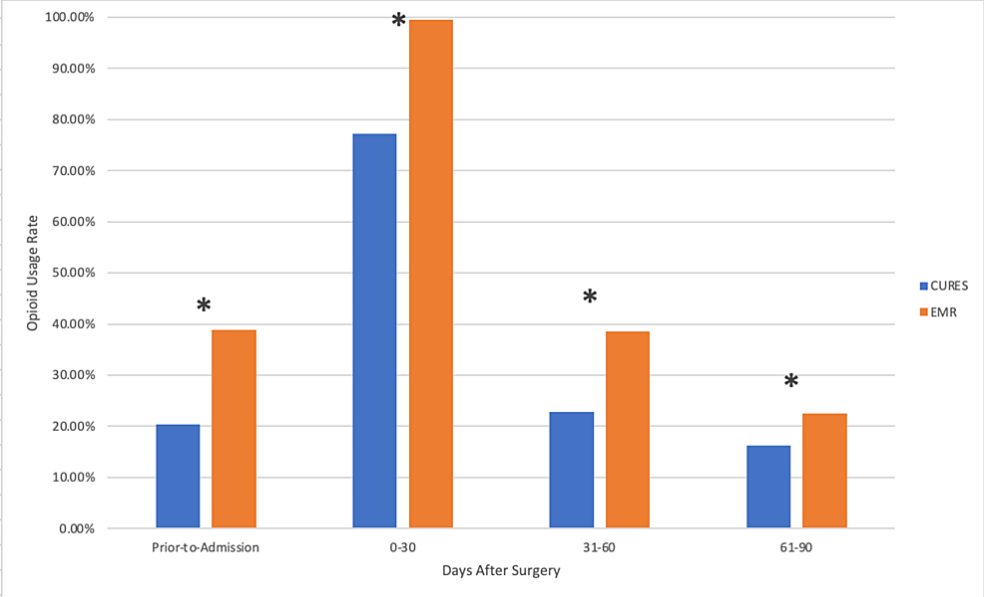
CURES utilization rate versus EMR prescription rate The image illustrates the observed CURES utilization rate as compared to the EMR prescription rate. CURES data represents when an active opioid prescription is utilized at a pharmacy. EMR data represents when a prescription is given out by a provider and logged in the medical records system. An asterisk signifies a significant discordance rate according to the 2 x 2 McNemar’s test (p-value < 0.05). CURES: Controlled Substance Utilization Review and Evaluation System; EMR: Electronic medical record.

According to CURES (Table 1), 75 patients (20.3%) had an active opioid prescription prior to their surgery. A total of 285 patients (77.2%) had an active opioid prescription between postoperative days 0-30; 84 patients (22.8%) had an active opioid prescription between postoperative days 31-60. Lastly, 60 patients (16.3%) had an active opioid prescription between postoperative days 61-90. At every time point, the EMR was more likely to report opioid use than CURES (Figure 1).

In the preoperative period (Table 2), there were 30 patients who were recorded in CURES having documented opioid prescriptions filled, but it was not reported in the EMR. Additionally, there were 98 instances of the EMR having documented opioid use and CURES having no documented administration. There was a 34.7% (128/369 records) discordancy rate observed in this period (p-value < 0.001) (Figure 1).

**Table 2:** CURES versus EMR discrepancies CURES: Controlled Substance Utilization Review and Evaluation System; EMR: Electronic medical record.

Postoperative Day	CURES - NO EMR - NO	CURES - YES EMR - YES	CURES - YES EMR - NO	CURES - NO EMR - YES	Discordance Rate (%)	p-values
Prior to admission	196	45	30	98	34.7%	<0.001
0-30	1	284	1	83	22.8%	<0.001
31-60	192	49	35	93	34.7%	<0.001
61-90	258	32	28	51	21.4%	<0.010

In the postoperative day 0-30 period (Table 2), there was one instance of CURES having documented opioid administration and the EMR having no documented opioid administration. On the other hand, there were 83 instances of the EMR having documented opioid administration but CURES having no documented opioid administration. This resulted in a 22.8% (84/369 records) discordancy rate observed in this period (p-value < 0.001) (Figure 1).

In the postoperative day 31-60 period (Table 2), there were 35 instances of CURES having recorded opioid administration and the EMR having no recorded opioid administration. There were 93 instances of the EMR having recorded opioid administration and CURES having no recorded opioid administration. This resulted in a 34.7% (128/369 records) discordancy rate in this period (p-value < 0.001) (Figure 1).

In the postoperative day 61-90 period (Table 2), there were 28 instances of CURES having recorded opioid administration and the EMR having no recorded opioid administration. There were 51 instances of the EMR having recorded opioid administration and CURES having no recorded opioid administration. This resulted in a 21.4% (79/369 records) discordancy rate in this period (p-value = 0.10) (Figure 1).

## Discussion

Our study found significant discordance between the opioid administration records observed in the CURES database and the hospital system EMR, with the EMR often reporting a higher rate of opioid prescription than what was recorded in CURES. The discordance was greatest and statistically significant for the first 60 days following surgery.

Prior studies have shown that orthopedic surgeons prescribe opioids at a higher rate than other specialties [8,9]. Our data collected from CURES showed that more than 75% of patients utilized an opioid prescription during the first month following surgery. This rate decreased to 22.8% in the second month and subsequently 16.3% in the third month following surgery. CURES data also shows that about 20% of patients had preoperative opioid prescription utilization. These rates are similar to those observed in other studies regarding opioid usage in following orthopedic surgery [10,11].

We do believe that CURES is the gold standard for opioid prescriptions as it truly records how many prescriptions are actually filled, while the EMR only records if the prescription was written [5]. In support of this, we found that the discrepancies between the EMR recording opioid use and CURES recording no opioid prescription are higher than CURES recording opioid utilization with no EMR in all time periods. This is likely due to opioid prescriptions being written that are never being utilized by patients. Alternatively, it could be that the EMR is not being reliably updated at each clinical encounter, and inactive prescriptions are still being reported. Relying solely on the EMR to determine opioid prescriptions will therefore skew the perception of the true opioid utilization rate and may over-report the rate of opioid usage. However, our study also identified multiple patients who receive opioids (CURES utilization) and who do not have a record of that prescription in the EMR. This could be due to a prescription being utilized later than when it was written or patients utilizing prescriptions from providers outside our hospital system’s EMR.

Multiple studies have established three problems in opioid prescribing habits within orthopedic surgery: Opioids tend to be prescribed in excess after procedures, there is a variation in the amount of opioids prescribed, and patients are not disposing of excess pills properly [12-14]. The EMR may not be the most current report of opioid use or patients may not be filling prescriptions that are prescribed since EMRs used for prescribing opioids only automatically record the prescription and not actual opioid utilization. As such, it is important to verify opioid use with the patient and ensure that the medical record is up to date, in addition to checking PDMP reporting.

We found that the EMR tends to over-report opioid prescription utilization and therefore opioid usage, which has clinical and research significance. There are several potential causes of this, but one could be that medical staff are not updating medication lists and not interviewing the patients regarding their opioid usage. This can bias the outcomes or conclusions of studies that solely utilize the EMR as a data source for opioid utilization. Additionally, clinical judgment may be skewed by solely utilizing the EMR to determine a patient’s opioid history. If a provider references only the EMR to decide if a patient is actively using an opioid prescription and should or should not have an opioid prescription renewed, then they may be under-prescribing or inadequately treating the patient’s pain up to 30% of the time. Conversely, if the prescriber only references their PDMP to make that description, there is a risk of overprescribing opioids up to 10% of the time.

The CDC states the need for established protocol within healthcare environments that requires providers to check PDMPs prior to prescribing opioids to maximize the effects of prescriptions and potentially decrease the risk of opioid addiction. Furthermore, according to federal policymakers and the Government Accountability Office, healthcare professionals must be required to use PDMPs when treating patients for them [15]. Underutilization is likely a contributing factor to the discrepancy between databases, which is why we recommend that providers utilize both databases as well as patient discussions, prior to prescribing opioids.

The PDMP has often been used to prevent “doctor shopping,” which occurs when patients seek opioid prescriptions from multiple providers, and this phenomenon is becoming more common among prescription drug users [1]. Among a population of postoperative orthopedic trauma patients, 20.8% of patients were discovered to have participated in “doctor shopping” for opioids [1]. It is important to develop programs that can help monitor and subsequently curb the “doctor shopping” practice that occurs among patients, especially in orthopedic trauma patients. This would allow for patients and their healthcare providers to have better-informed conversations about patient access to opioids and, if necessary, initiate conversations around harmful substance use and develop subsequent care plans for these patients.

Our study did have several limitations that should be addressed. First, this was a single-center study, and the findings of our center may not be generalizable to all centers. There may be differences in discordance found in quality improvement studies done at other institutions, but assessing the discordance is important to have a holistic understanding of opioid prescription preceding and the following surgery. Second, we are limited by the restraints in the CURES system opioid prescription data in the year preceding the observation date, which limited the size of our cohort, given the retrospective nature of the study. CURES is also limited in being a single-state database, meaning out-of-state utilizations are not captured within the database. Furthermore, PDMPs record the date an opioid prescription is filled. This date is not necessarily the same as the date of prescription that might be listed in the EMR; this might have caused some errors in our analysis due to prescriptions that were given and filled in two different time periods of our study. Another limitation is that we recorded opioid use in a binary fashion rather than observing the milligram morphine equivalents (MMEs) listed in both databases; this could have led to an under-reporting of the true discrepancy.

The retrospective nature of this study further limited our ability to verify medication lists with the patients themselves, but this very limitation sheds light on the dangers of relying solely on the EMR to retrospectively evaluate a patient’s medication usage. The medication list in the EMR is meant to be updated with each patient encounter, often by a pharmacist, nurse, allied health professional, or physician. When the EMR is reviewed, we often assume that the medication list reflects what medication the patient is currently taking; however, the fact that the EMR often over-reported opioid use compared to opioids that were actually prescribed in the state of California suggests that the medication list is not always accurately updated.

## Conclusions

We found high discordance rates between a state database and an in-house medical record system preoperatively and postoperatively. While the PDMP is often considered to be the gold standard for opioid utilization, there was a significant proportion of CURES prescriptions that were not reflected in the EMR, which is often the primary method that many clinicians use for determining opioid usage. Neither database may truly reflect a patient’s opioid usage. We recommend that both the EMR and state PDMP should be reviewed with the patient to capture a patient’s opioid use most accurately, and the EMR should subsequently be updated based on true utilization rates. Understanding prescribing patterns is essential to effectively manage patients’ pain and prevent opioid mismanagement.
